# Quantum Biochemistry Insights into Ligand Recognition at the a_1A_-Adrenoceptor

**DOI:** 10.1021/acsomega.5c08861

**Published:** 2026-02-11

**Authors:** Luana Talinne da Costa Gomes, Katyanna Sales Bezerra, Elaine Cristina Gavioli, Jonas Ivan Nobre Oliveira, Douglas Soares Galvão, Umberto Laino Fulco, Edilson Dantas da Silva Junior

**Affiliations:** † Department of Biophysics and Pharmacology, 28123 Federal University of Rio Grande Do Norte, Natal 59072-970, Rio Grande do Norte, Brazil; ‡ Applied Physics Department, University of Campinas, Campinas, São Paulo 13083-859, Brazil

## Abstract

Understanding the molecular basis of ligand recognition at α_1A_-adrenoceptor (α_1A_-AR) is essential for developing highly selective therapeutic agents. In this study, we applied a quantum biochemistry approach combining density functional theory (DFT) with the molecular fractionation with conjugate caps (MFCC) method to perform a detailed energetic characterization of the interactions between α_1A_-AR and three ligands with distinct pharmacological profiles: the endogenous nonselective agonist noradrenaline, the partial and selective α_1A_-AR agonist oxymetazoline, and the selective α_1A_-AR antagonist tamsulosin. Our calculations of total binding energy accurately reproduced the experimental relative affinity ranking (tamsulosin > oxymetazoline > noradrenaline), supporting the reliability of the MFCC-DFT protocol in modeling receptor–ligand interactions at quantum resolution. A total of 81, 88, and 93 amino acid residues of α_1A_-AR interacted with noradrenaline, oxymetazoline, and tamsulosin, respectively. The most energetically relevant residues were located within 4 Å. A comprehensive residue-level analysis revealed that ASP106, VAL107, PHE288, and PHE312 are key contributors to the total binding energy of all ligands, corroborating evidence from structural and mutagenesis studies. Specifically for oxymetazoline, this ligand contains a *tert*-butyl group that establishes nonpolar interactions with residues such as VAL185 and ALA189, which are not observed in the noradrenaline-α_1A_-AR complex. Additionally, unlike noradrenaline, oxymetazoline exhibits an attractive interaction with MET292 and does not engage in polar interactions with SER188. These differential interaction patterns may contribute to the distinct pharmacological profile of oxymetazoline relative to noradrenaline. Tamsulosin also exhibited a distinct interaction profile compared to agonists noradrenaline and oxymetazoline, interacting with residues located in the extracellular vestibule, including SER83, PHE86, GLU87, TRP102, CYS176, and LYS309. These additional interactions play a pivotal role in stabilizing tamsulosin within the binding pocket, contributing to its high selectivity and antagonistic behavior at the α_1A_-AR. Altogether, these findings provide a robust theoretical framework for understanding the molecular determinants of functional selectivity and subtype specificity at α_1A_-AR, offering valuable insights for the rational design of new ligands with improved selectivity, efficacy, and safety profiles.

## Graphical abstract

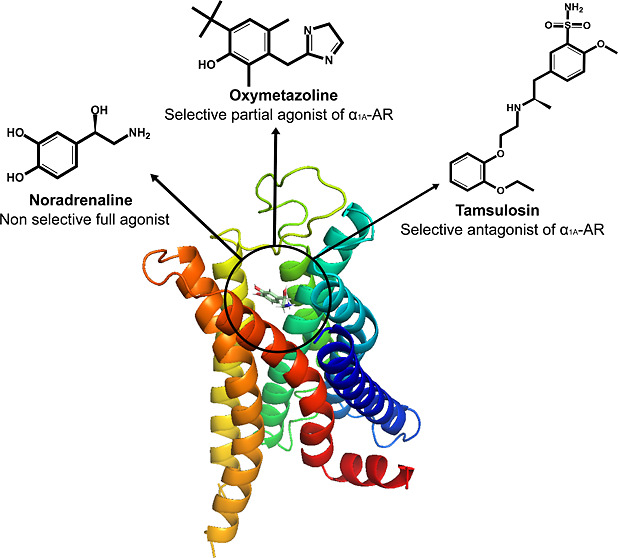

## Introduction

Adrenoceptors (ARs) are G-protein coupled receptors that mediate the physiological effects of the endogenous catecholamines noradrenaline and adrenaline. Based on their amino acid sequence, pharmacological profile, and functional properties, these receptors are classified into three distinct families: α_1_-, α_2_-, and β-ARs. Each adrenoceptor family is further subdivided into specific subtypes: α_1A_, α_1B_, and α_1D_ for the α_1_-ARs family; α_2A_, α_2B_, and α_2C_ for the α_2_-ARs family; and β_1_, β_2_, and β_3_ for the β-ARs family.^1–5^

The α_1_-ARs are widely distributed across central nervous structures and peripheral organs, notably within the cardiovascular, hepatic, and genitourinary systems, with each subtype exhibiting a distinct tissue distribution that contributes to specific physiological responses. In this context, α_1A_-AR subtype stands out due to its prominent role in regulating vascular and genitourinary smooth muscle activity, cardiac function, and neurophysiological processes, particularly those related to memory and cognition.^1,3–5^ In peripheral tissues, the activation of postsynaptic α_1A_-ARs induces smooth muscle contraction in densely innervated small caliber arteries, prostate, ureter, urethra, and other genitourinary organs.^6^ This mechanism is clinically exploited, as demonstrated by the α_1A_-AR agonist oxymetazoline, which is used to alleviate nasal congestion through vasoconstriction in the nasal mucosa.^7,8^ Conversely, the α_1A_-AR antagonist tamsulosin is used to manage benign prostatic hyperplasia as it relaxes the smooth muscle of the prostate and urethra, thereby improving urine flow.^7,9^

Preclinical studies also suggest potential therapeutic effects for α_1A_-AR agonists for the treatment of heart failure and ischemia and mental disorders related to mnemonic deficits. In fact, stimulation of α_1A_-ARs with agonists may exert cardioprotective effects, including enhanced contractility in failing hearts, protection against ischemic injury, antiapoptotic effects, and promotion of adaptive hypertrophy.^4,10,11^ Moreover, systemic overexpression of α_1A_-ARs in transgenic mice, as well as pharmacological stimulation of the α_1A_-ARs with cirazoline, enhances cognition, suggesting that α_1A_-ARs activation could represent a novel therapeutic strategy for cognitive deficits of Alzheimer’s disease.^4,12^ Stimulation of α_1A_-ARs has also been implicated in stress-induced memory formation and consolidation. Thus, α_1A_-AR antagonism may offer psychotherapeutic benefits for post-traumatic stress disorder (PTSD).^4^

The limited specificity of currently available ligands for α_1A_-ARs poses a significant challenge for both basic research and therapeutic applications. Although some compounds demonstrate preferential selectivity for the α_1A_-AR subtype, many also interact substantially with other adrenergic subtypes or unrelated receptor families, compromising their efficacy and safety profiles.^13,14^ For instance, oxymetazoline exhibits considerable α_1A_-AR selectivity, but also functions as an agonist at α_2_-AR and serotoninergic receptors (5-HT_1A/B/D_).^1^ Similarly, tamsulosin, originally developed as a selective antagonist for α_1A_-ARs, displays comparable potency at α_1D_-ARs and also interacts with 5-HT_1A_ and dopaminergic receptors (D_2_ and D_3_).^1,15,16^ This lack of selectivity not only increases the risk of off-target effects but also impairs the precise characterization of physiological and therapeutic responses mediated by α_1A_-ARs.^13,14^

Understanding the structural basis of these selectivity limitations requires detailed examination of the molecular determinants governing ligand recognition at the α_1A_-AR. The molecular basis of ligand recognition and signaling at the α_1A_-AR is governed by specific interactions within its orthosteric binding pocket. The composition of amino acid residues forming this site dictates the intermolecular interactions that enable ligand recognition and stabilization, making detailed analysis of individual residue contributions essential to elucidate the functional plasticity of ligands and their coupling mechanisms.^17^ Structural studies have identified key residues critical for α_1A_-AR function.^5,14^ For instance, ASP106 is an evolutionarily conserved residue that plays a fundamental role in ligand recognition across all adrenoceptor subtypes.^5^ Its functional relevance is underscored by mutational analyses demonstrating that alanine substitution at this position abolishes binding of the α_1_-AR antagonist prazosin.^18^

Additional structural determinants include PHE288 and PHE312, both identified in cryo-EM structures as crucial for ligand recognition. PHE288 displays high conservation across adrenoceptor subtypes, suggesting a fundamental structural role in maintaining binding pocket architecture. In contrast, PHE312 is conserved among α-ARs but differs in β-ARs, potentially contributing to subtype selectivity.^5,14,19^ Functional studies confirm this distinction, as PHE312 mutation significantly reduces binding affinity for several antagonists and imidazoline-type agonists like oxymetazoline, cirazoline, and clonidine, while sparing affinity for phenethylamine-type agonists including epinephrine, methoxamine, and phenylephrine^20^

To address the challenges of limited ligand selectivity at α_1A_-ARs, we performed computational calculations using density functional theory (DFT) framework combined with molecular fractionation with conjugate caps (MFCC) approach. This methodology was used to investigate the interactions of three ligands (noradrenaline, oxymetazoline, and tamsulosin) with the Cryo-EM structures of the α_1A_-AR. By analyzing these structural data, we quantified the interaction energy of individual amino acid residues involved in ligand binding. This approach provided detailed insights into the molecular determinants of ligand selectivity and receptor interaction, offering a solid basis for the rational design of more selective α_1A_-AR ligands.

## Materials and Methods

Computational analyses were performed using Cryo-EM structures of the α_1A_-AR bound to noradrenaline, oxymetazoline, and tamsulosin (Figure 1). These complexes were retrieved from the Protein Data Bank (PDB) under the following identification codes: 7YMH, 7YM8, and 7YMJ, respectively.^5^ First, the protonation states of both the ligands and the amino acid residues of the protein at pH 7.5 (pH used in each Cryo-EM structures) were determined using MarvinSketch version 17.24 (Marvin Beans Suite–ChemAxon) and the PROPKA 3.1 software package, respectively.^21^ Hydrogen atoms and side chains of amino acids that were missing from the Cryo-EM structures due to the intrinsic resolution limitations of the technique were manually added. Then, amino acid main-chain atoms were maintained fixed while side-chains were subjected to classical geometry optimization using the CHARMM (Chemistry at Harvard Molecular Mechanics) force field, a process that refines the atomic positions to minimize energy.^22^ Convergence criteria included a total energy change threshold of 10^–5^ kcal·mol^–1^, a root-mean-square gradient below 10^–3^ kcal·mol^–1^, and a maximum atomic displacement of less than 10^–5^ Å. The illustration presented in Figure S1 (Supporting Information) shows the comparison between the experimental data and the geometric optimization performed using the CHARMM force field.

**Figure 1:**
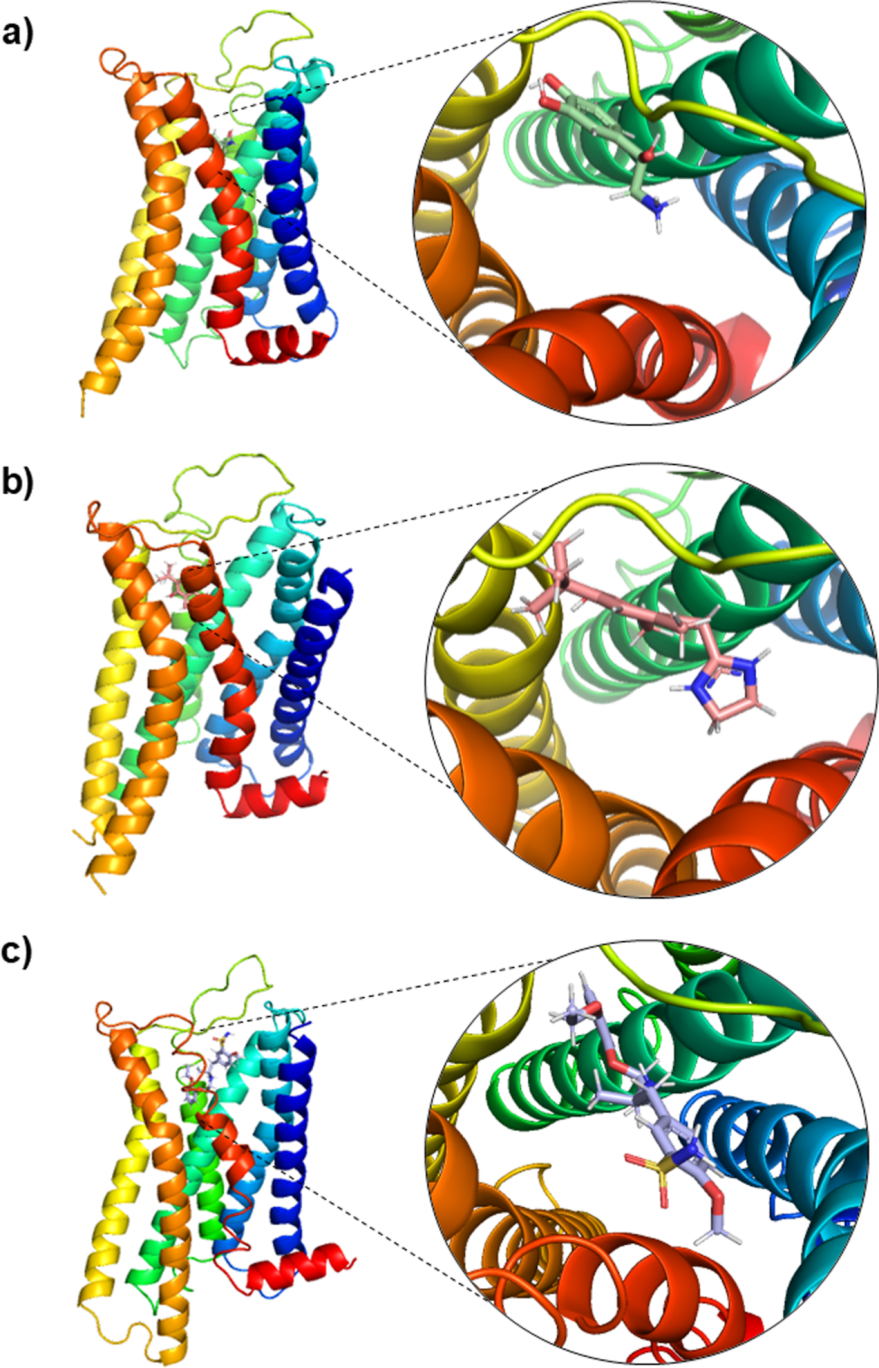
Representation of the Cryo-EM structures of the α_1A_-adrenoceptor bound to (a) noradrenaline, (b) oxymetazoline, and (c) tamsulosin. The overall receptor structure is shown as a rainbow-colored cartoon. Magnified views highlight the binding pockets of each ligand within the orthosteric site.

The binding energy between the ligands and α_1A_-AR was determined by using the MFCC method within the DFT framework. The MFCC scheme has been extensively applied in calculating binding energies in protein–ligand, and protein–protein complexes.^17,22–29^ This technique enables the analysis of numerous amino acids in a protein with a low computational demand, which is essential in the study of complex biological systems. It was originally developed as a fractionation strategy to facilitate efficient ab initio calculations by decomposing the interaction energy among protein molecules into individual amounts of interaction that can be efficiently computed.^30,31^ In this context, the protein is fragmented into amino acid residues by cleaving the covalent bonds between adjacent residues (peptide bonds), and hydrogen atoms are added to preserve the valence of each resulting fragment. Subsequently, the interaction energy between the ligand (*L*) and the *i*th amino acid residue (*R*_*i*_) is calculated according to the following equation

1 $$\begin{array}{lll} E\left(L-R_{i}\right) & = & E\left(L-C_{i-1}R_{i}C_{i+1}\right)-E\left(L-C_{i-1}C_{i+1}\right)\\ & - & E\left(C_{i-1}R_{i}C_{i+1}\right)+E\left(C_{i-1}C_{i+1}\right) \end{array}$$

In the equation above, the first term, *E*(*L*–*C*_*i*–1_ *R*_*i*_ *C*_*i*+1_), represents the total energy of the system comprising the ligand and the capped residue. The second term, *E*(*L*–*C*_*i*–1_ *C*_*i*+1_), corresponds to the total energy of the ligand with the caps alone. The third term, *E*(*C*_*i*–1_ *R*_*i*_ *C*_*i*+1_), denotes the total energy of the residue along with its capping groups, whereas the fourth and final term, *E*(*C*_*i*–1_ *C*_*i*+1_), accounts for the energy of the caps with hydrogen atoms added to saturate the dangling bonds.

Energetic calculations for individual residues within the binding site were carried out using the Gaussian G09 software package, applying DFT formalism under the generalized gradient approximation (GGA) with the B97D functional.^32,33^ This functional incorporates dispersion corrections, improving the characterization of noncovalent interactions. In order to describe the electronic wave function and expand the Kohn–Sham orbitals, taking into account all the electrons in the system, we chose to use the 6–311 + G­(*d, p*) basis set, which includes triple-ζ valence functions, additional polarization components (*d, p*) and a diffuse function (+).

An accurate representation of the electrostatic field is essential in theoretical investigations involving biomolecules. Although the relative electrical permittivity of proteins is around 4, in an aqueous medium, this value can reach 78. Previous studies using the MFCC method evaluated different values of this property, concluding that ε = 40 promotes a good correlation between computational results and experimental data.^34,35^ Thus, we applied the polarizable continuous conductor model (CPCM)^36^ considering values of ε equal to 10 and 40 to describe the medium around the fragments obtained via MFCC. The choice of these values is not arbitrary. Studies such as that of Vicatos et al.^37^ have demonstrated that adjusting the ε parameter to 40 significantly improves the estimation of protein stability, promoting greater correspondence between theoretical results and empirical data. Similarly, Morais et al.^38^ analyzed homogeneous and heterogeneous dielectric approaches, confirming that ε = 40 is appropriate to model the electrostatic field present at the interface between proteins in biomolecular complexes. Although the dielectric constant ε = 10 may slightly overestimate the absolute binding energies, it provides a more realistic representation of the internal electrostatic conditions of the receptor, approximating the nonpolar microenvironment of the α_1A_-AR binding site compared to higher dielectric media. Furthermore, the overall binding energy trends and interaction profiles of the residues remained consistent when recalculated with ε = 40, confirming that the conclusions are robust and not significantly influenced by the dielectric parameter. This consistency reinforces that the observed interaction patterns primarily reflect intrinsic physicochemical characteristics of ligand recognition by α_1A_-AR.

Furthermore, we emphasize that the main objective of this work is not to reproduce absolute binding energies with quantitative precision, but to identify and characterize the essential amino acid residues that contribute most significantly to ligand recognition and affinity at the α_1A_-AR receptor binding site. It is also important to note that entropic effects were not included in the calculations presented, which partially explains the deviation from the experimental energy magnitudes.

To ensure that no relevant interaction was overlooked, a convergence analysis of binding energy as a function of binding site radius was performed. This procedure defined an appropriate limit for the number of amino acid residues included in the calculations. Specifically, the individual interaction energies of residues within successive concentric spheres centered on the ligand were incrementally summed. The binding site radius (r) was defined as half the incremental distance (*r* = R/2, with *R* = 1, 2, 3, 4, ..., in Ångströms), so the radius increased by 0.5 Å at each step of the analysis. Smaller values of r correspond to residues in direct contact with the ligand, while larger values include medium- and long–range interactions within the binding site. Convergence was considered achieved when the variation in total interaction energy between two consecutive radii was less than 10%.

## Results and Discussion

To facilitate the analysis of binding energy and intermolecular interactions, the ligands were divided into distinct regions according to their chemical groups, as follows: noradrenaline–catechol ring (region (i) and β -hydroxyethylamine group (region (ii) (Figure 2a, left panel); oxymetazoline–imidazoline group (region (i), dimethylphenol group linked by a methylene bridge (region (ii), and *tert*-butyl group (region (iii) (Figure 2b, left panel); tamsulosin–ethoxyphenoxy group (region (i), ethylaminopropyl group (region (ii), and methoxybenzenesulfonamide group (region (iii) (Figure 2c, left panel).

**Figure 2:**
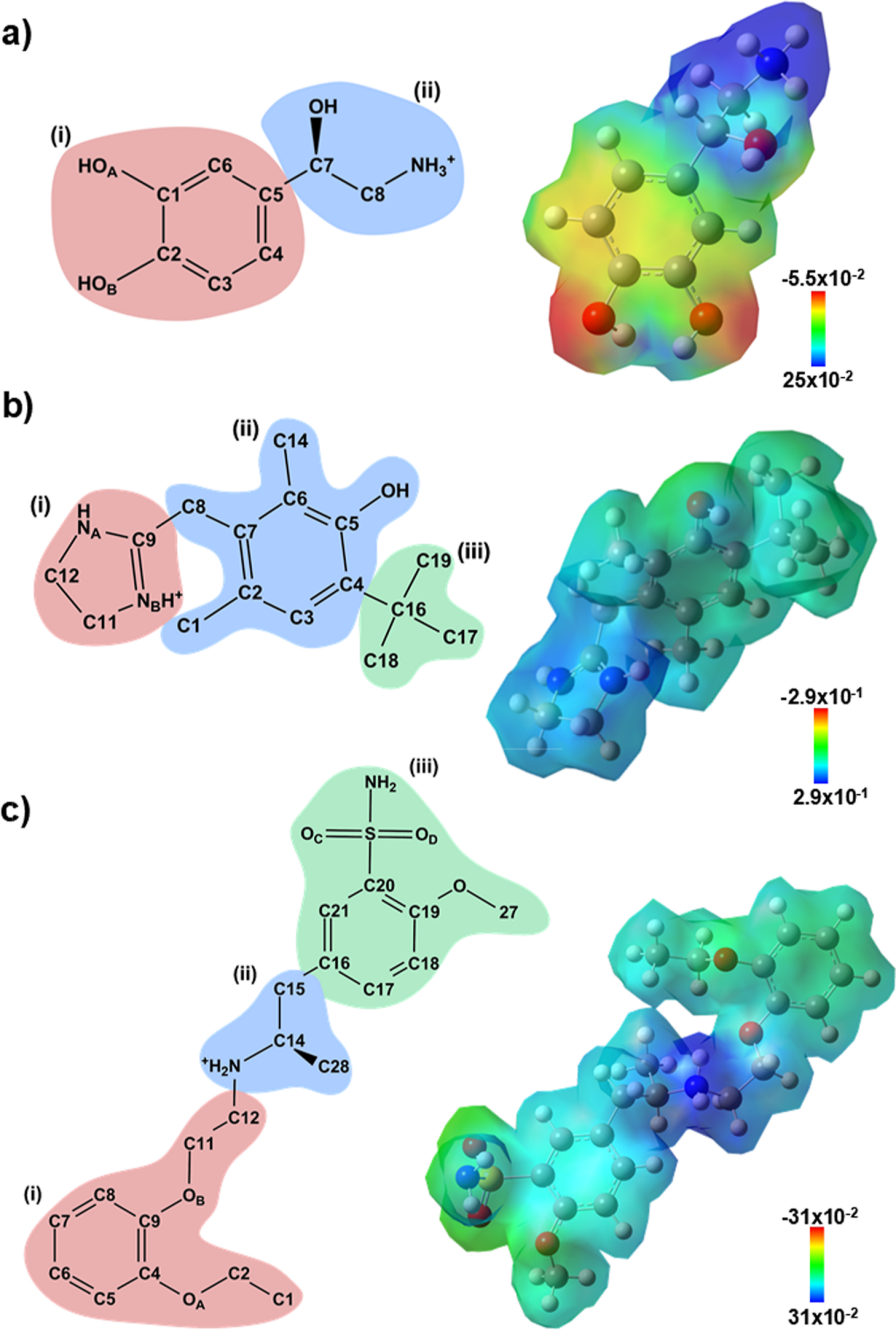
Schematic representations of the chemical structures of the ligands are subdivided into distinct regions, along with their respective DFT-derived electronic densities projected onto electrostatic potential (ESP) isosurfaces. (a) Noradrenaline; (b) oxymetazoline; and (c) tamsulosin. The colored regions in the 2D structures correspond to chemically distinct groups considered for interaction analysis. The ESP maps illustrate the charge distribution across each ligand surface, with the color scale indicating the range of electrostatic potential values (in atomic units).

Considering the pH 7.5 of the Cryo-EM structures, noradrenaline, oxymetazoline, and tamsulosin exhibited a partial positive charge (+1) on the nitrogen atom of the β-hydroxyethylamine, imidazoline, and ethylaminopropyl groups, respectively (Figure 2, left panels). Electrostatic potential maps revealed that the ligands displayed an electropositive character in the regions containing the protonated nitrogen (bluish tones) (Figure 2, right panels). Additionally, noradrenaline exhibited a more prominent electronegative nature at the hydroxyl groups of the catechol ring (reddish tone) (Figure 2a, right panel). In contrast, regions ii and iii of oxymetazoline and regions i and ii of tamsulosin displayed neutral characteristics (greenish tone) (Figure 2b,c, respectively; right panels).

The binding interaction energies and convergence criteria were individually assessed for the agonists noradrenaline and oxymetazoline, as well as the antagonist tamsulosin. Each ligand was defined according to the radius of its binding pocket (expressed in Ångströms) and the corresponding interaction energy (reported in kcal·mol^–1^). To gain deeper insight into the molecular interactions involved in the formation of α_1A_-AR–ligand complexes, it is essential to account for all amino acid residues that contribute either attractive or repulsive forces within the binding environment. These residues can significantly affect the overall interaction energy and, thus, the pharmacological potential of each ligand. Mapping the energy contributions within the binding site provides a theoretical basis for structure-guided ligand optimization, offering details that can subsequently support the rational design of α_1A_-AR modulators with enhanced affinity and selectivity.^17,39^

The convergence analysis of the ligand–receptor complexes was conducted by calculating the total binding energy. This energy was derived from the sum of individual interaction energies between the ligands and amino acid residues from each tested binding pocket radius. Both attractive (negative) and repulsive (positive) interactions were considered in this calculation. This analysis enabled the determination of an optimal binding pocket radius, defined as the point at which the interaction energies between the ligands and the residues within a given radius exhibited minimal variation, characterized by less than 10% of the total binding energy. From this radius onward, the energy values remained stable, indicating convergence. Moreover, this approach also allowed the identification of the amino acid residues that contribute most significantly to the ligand–receptor interaction, providing a basis for robust comparison of the binding profiles of different ligands for a given receptor.^17,40,41^

The total binding energy for noradrenaline/α_1A_-AR, oxymetazoline/α_1A_-AR, and tamsulosin/α_1A_-AR complexes is shown in Figure 3 as a function of the radius r, considering dielectric constants ε = 10 and ε = 40. The energetic convergence of the systems was attained within a radius of 5 Å for the noradrenaline/α_1A_-AR complex, and 4 Å for oxymetazoline/α_1A_-AR and tamsulosin/α_1A_-AR complexes at both dielectric constants. Nonetheless, all the calculations were performed up to a radius of *r* = 10 Å to ensure the evaluation of all relevant residues involved in the ligand–receptor interactions, encompassing a total of 81, 88, and 93 amino acid residues interactions for noradrenaline, oxymetazoline, and tamsulosin, respectively (Tables S1, S2, and S3 respectively; Supporting Information).

**Figure 3:**
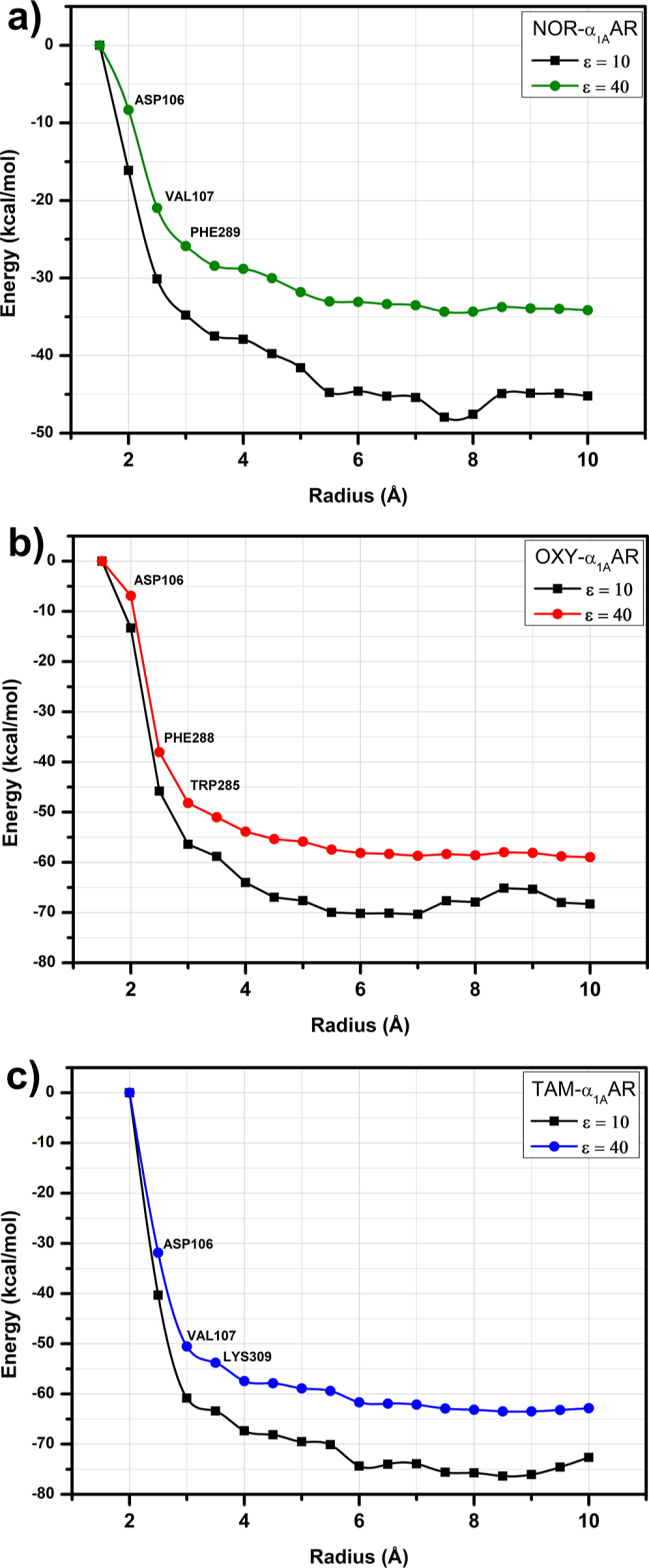
Representation of the total interaction energy of the α_1A_-adrenoceptor in complex with (a) noradrenaline, (b) oxymetazoline, and (c) tamsulosin as a function of the ligand pocket radius r calculated using the functional GGA B97D within the MFCC scheme using two dielectric constants (ε = 10 and ε = 40). The highlighted amino acid residues are those responsible for the largest binding energies.

The total binding energy of ligands at α_1A_-AR was −45.1 kcal·mol^–1^ (ε = 10) and −34.1 kcal·mol^–1^ (ε = 40) for noradrenaline (Figure 3a), −68.3 kcal·mol^–1^ (ε = 10) and −58.9 kcal·mol^–1^ (ε = 40) for oxymetazoline (Figure 3b), and −72.3 kcal·mol^–1^ (ε = 10) and −62.6 kcal·mol^–1^ (ε = 40) for tamsulosin (Figure 3c). The largest energy variations were observed in the 2.0–3.0 Å range for the noradrenaline/α_1A_-AR and oxymetazoline/α_1A_-AR complexes, primarily due to strong attractive interactions between noradrenaline and residues ASP106, VAL107, and PHE289 (Figure 3a), as well as between oxymetazoline and residues ASP106, PHE228, and TRP285 (Figure 3b). In the tamsulosin/α_1A_-AR complex, the most pronounced energy variation occurred between 2.5 and 3.5 Å, mainly due to contributions from residues ASP106, PHE312, VAL107, and LYS309 (Figure 3c).

It is also noteworthy that the rank order of total binding energy strength at α_1A_-AR identified in our in silico analysis was tamsulosin > oxymetazoline > noradrenaline. This is consistent with the experimentally determined affinity (pKi) values for these ligands at α_1A_-AR, which are tamsulosin (pKi 9.4–10.7) > oxymetazoline (pKi 7.2–8.2) > noradrenaline (pKi 4.8–6.4).^2^ These data supported the notion that the total binding energies calculated via quantum biochemistry analysis using the MFCC-DFT approach can be reliably correlated with experimentally determined ligand affinities at receptors, thereby establishing MFCC-DFT as a powerful computational tool in molecular recognition and rational drug design.^42–44^

In all Cryo-EM structures of the α_1A_-AR, the chemical interactions (attractive or repulsive) between each ligand and the amino acid residues of the binding pocket showed no discrepancies between dielectric constants ε = 10 and ε = 40, which reinforces the consistency of the ligand-α_1A_-AR binding patterns (see Tables S1, S2, and S3 in Supporting Information). Moreover, studies employing the MFCC method combined with DFT have demonstrated that simulations performed using a dielectric constant of ε = 40 produce results that show better agreement with experimental data.^22,35,40,45,46^ In view of this, all subsequent discussions will be based on the results obtained from our simulations considering only the dielectric constant ε = 40.

Figure 4 presents schematic representations of the interaction energies between the noradrenaline/α_1A_-AR complex (Figure 4a), oxymetazoline/α_1A_-AR complex (Figure 4b), and tamsulosin/α_1A_-AR complex (Figure 4c). The horizontal bars indicate, quantitatively, the interaction energy values (in kcal·mol^–1^) for each amino acid, allowing a clear visualization of the magnitude and relevance of each interaction. The left portion of each graph highlights the most relevant residues for the stabilization of the complexes, as well as the regions and atoms of the ligands located in their immediate proximity to the active site. On the right, the respective interaction radii (in Å) are reported, which indicate the distance between the residues and the ligands.

**Figure 4:**
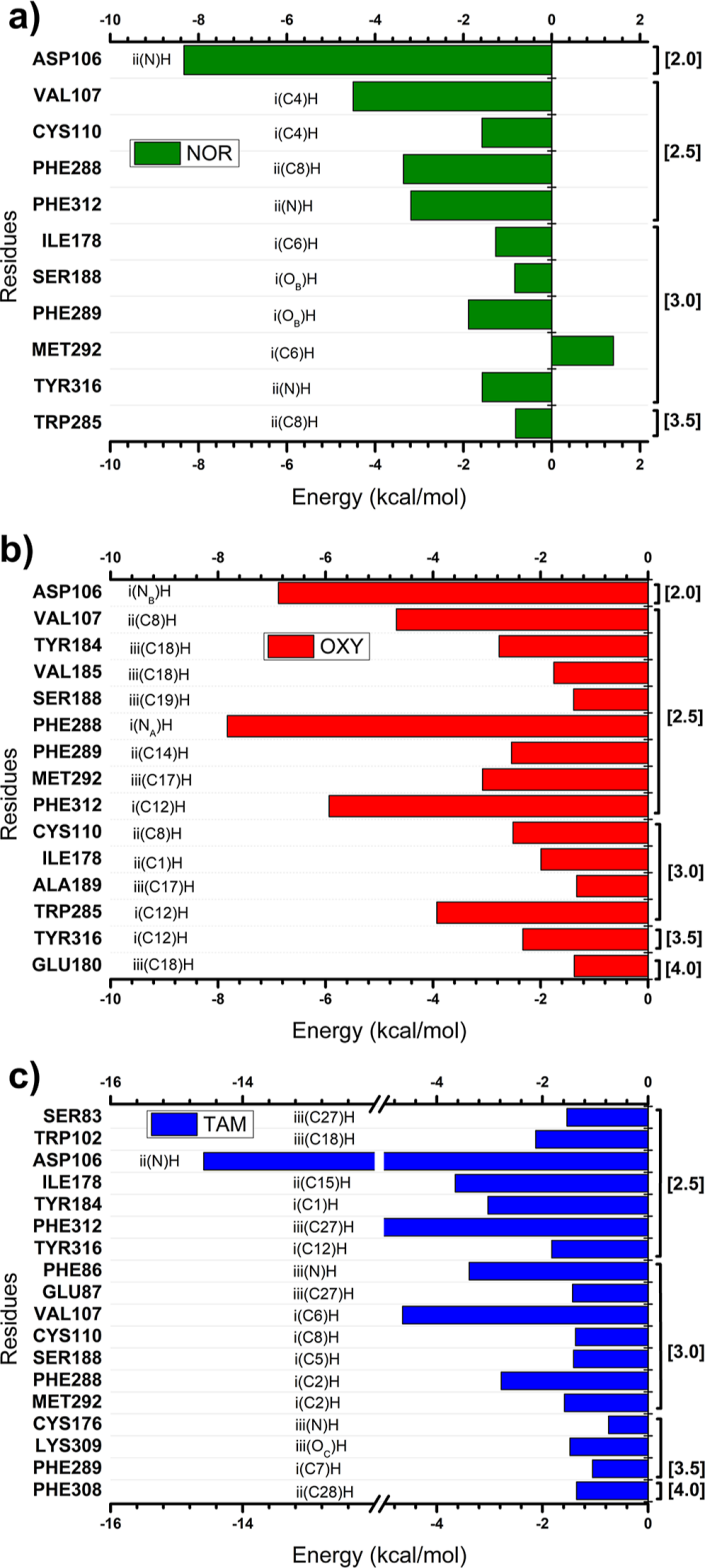
Graphic panels showing the most relevant residues contributing to the total binding energy in the three evaluated complexes: (a) noradrenaline-α_1A_-adrenoceptor (green); (b) oxymetazoline-α_1A_-adrenoceptor (red); and (c) tamsulosin-α_1A_-adrenoceptor (blue). For each residue, the interacting ligand regions and atoms within the binding pocket are indicated, along with the minimum distances between the residue and the corresponding ligand atoms.

In the noradrenaline/α_1A_-AR complex (Figure 4a), the residues that contributed most to the interaction energy with the ligand (in kcal·mol^–1^) were, in descending order: ASP106 (−8.32) > VAL107 (−4.50) > PHE288 (−3.36) > PHE312 (−3.19) > PHE289 (−1.89) > CYS110 (−1.58) > TYR316 (−1.57) > ILE178 (−1.27) > SER188 (−0.84) > TRP285 (−0.82) > MET292 (1.40). The catechol ring (region (i) was involved in six interactions (VAL107, CYS110, ILE178, SER188, PHE289, and MET292), contributing to an interaction energy of −8.68 kcal·mol^–1^. Meanwhile, the β-hydroxyethylamine group (region (ii) was associated with five interactions (ASP106, PHE288, PHE312, TYR316, and TRP285), contributing −17.26 kcal·mol^–1^ to the overall interaction energy. The presence of a protonated nitrogen atom in region ii facilitates electrostatic interactions with neutral or negatively charged residues, accounting for its higher contribution to the total interaction energy relative to region i.

The residues with the greatest contributions to the interaction energy (in kcal·mol^–1^) in the oxymetazoline/α_1A_-AR complex (Figure 4b), listed in descending order, were: PHE288 (−7.83) > ASP106 (−6.88) > PHE312 (−5.93) > VAL107 (−4.68) > TRP285 (−3.93) > MET292 (−3.08) > TYR184 (−2.77) > PHE289 (−2.54) > CYS110 (−2.51) > TYR316 (−2.33) > ILE178 (−1.99) > VAL185 (−1.75) > SER188 (−1.39) > GLU180 (−1.37) > ALA189 (−1.33). The imidazoline group (region (i), comprising interactions with ASP106, TRP285, PHE288, PHE312, and TYR316, accounted for the largest interaction energy (−26.9 kcal·mol^–1^), likely due to the presence of a protonated nitrogen atom that promotes strong electrostatic interactions with the surrounding residues. The dimethylphenol group linked by a methylene bridge (region (ii) interacted with VAL107, CYS110, ILE178, and PHE289, contributing −11.7 kcal·mol^–1^ to the total interaction energy. Additionally, the *tert*-butyl group (region (iii) established interactions with GLU180, TYR184, VAL185, SER188, ALA189, and MET292, also contributing −11.7 kcal·mol^–1^.

In the tamsulosin/α_1A_-AR complex (Figure 4c), the residues contributing most significantly to the interaction energy (in kcal·mol^–1^), in descending order, were: ASP106 (−14.59) > PHE312 (−5.08) > VAL107 (−4.65) > ILE178 (−3.65) > PHE86 (−3.39) > TYR184 (−3.03) > PHE288 (−2.78) > TRP102 (−2.13) > TYR316 (−1.83) > SER83 (−1.54) > MET292 (−1.58) > LYS309 (−1.48) > GLU87 (−1.43) > SER188 (−1.41) > CYS110 (−1.38) > PHE308 (−1.35) > PHE289 (−1.05) > CYS176 (−0.75). The ethoxyphenoxy group (region (i) formed interactions with VAL107, CYS110, TYR184, SER188, PHE288, MET292, PHE289, and TYR316, contributing −17.7 kcal·mol^–1^ to the total interaction energy. The ethylaminopropyl moiety (region (ii), associated with ASP106, ILE178, and PHE308, accounted for the largest energetic contribution among the three regions (−19.6 kcal·mol^–1^), likely due to electrostatic interactions involving its protonated amine. The methoxybenzenesulfonamide group (region (iii) interacted with SER83, PHE86, GLU87, TRP102, CYS176, LYS309, and PHE312, contributing −15.8 kcal·mol^–1^.

Among the key amino acid residues contributing most to the interaction energy in the three Cryo-EM complexes (Figure 4), ten were consistently involved in ligand binding across all ligands: ASP106, VAL107, CYS110, ILE178, SER188, MET292, PHE288, PHE289, PHE312, and TYR316, highlighting their potential functional relevance in stabilizing ligand-α_1A_-AR interactions. Additionally, TRP285 was an important contributor to the interaction energy of both noradrenaline and oxymetazoline with the α_1A_-AR. Conversely, TYR184 was a relevant common residue contributing to the interaction energy of both oxymetazoline and tamsulosin with the α_1A_-AR. GLU180, ALA189, and VAL185 significantly contributed to the interaction energy in oxymetazoline binding to the α_1A_-AR, while their energetic contribution was less pronounced in the binding of noradrenaline and tamsulosin to the α_1A_-AR. In the tamsulosin/α_1A_-AR complex, unlike in the noradrenaline- or oxymetazoline-bound α_1A_-AR complexes, SER83, PHE86, GLU87, TRP102, CYS176, PHE308, and LYS309 were identified as energetically relevant residues.

All three ligands exhibited high interaction energy with ASP106, which showed the highest energetic contribution for both noradrenaline and tamsulosin and the second highest for oxymetazoline. The strong attraction of noradrenaline, oxymetazoline, and tamsulosin to ASP106 can be attributed to electrostatic interactions, which are characterized by relatively high energetic contributions compared to other noncovalent forces.^47^ Specifically, the interaction between the noradrenaline and ASP106 involves hydrogen bonds (H-bond), salt bridges, and a π-cation interaction (Figure 5a), all mediated by the protonated nitrogen atom located in ligand region ii. Similarly, the protonated nitrogen atom in region i of oxymetazoline and region ii of tamsulosin interacts with ASP106 through H-bonds and salt bridges (Figures 6a and 7a). Consistent with our findings, previous studies have shown that the interaction between adrenergic ligands and ASP106 in the α_1A_-AR primarily involves electrostatic forces.^14,18^

**Figure 5:**
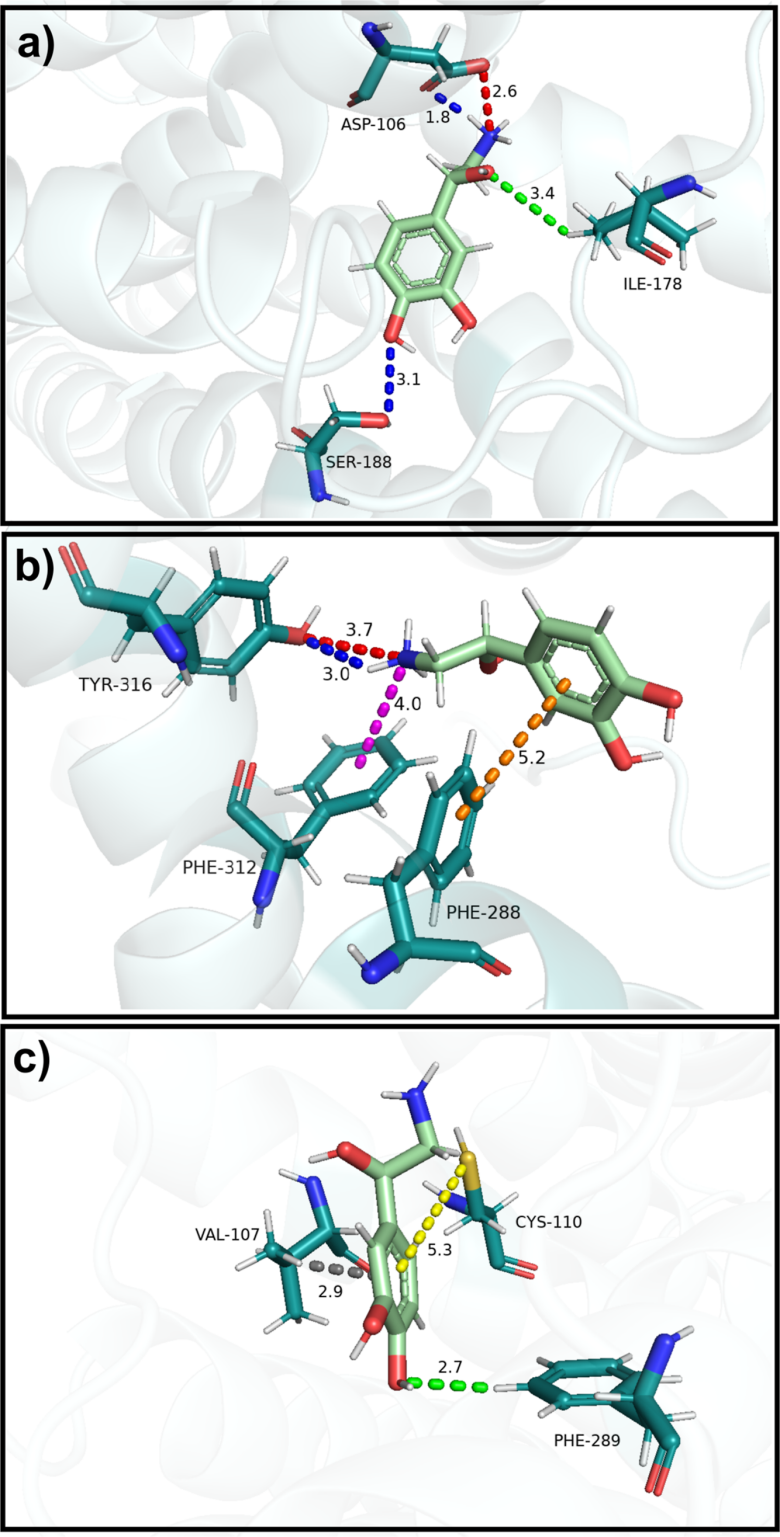
Visual representation of some of the main intermolecular interactions between noradrenaline and the α_1A_-adrenoceptor. (a) Noradrenaline interactions with ASP106, ILE178, and SER188. (b) Noradrenaline interactions with PHE288, PHE312, and TYR316. (c) Noradrenaline interactions with VAL107, CYS110, and PHE289. Dashed lines represent different types of interactions: salt bridge (red), hydrogen bond (blue), nonconventional hydrogen bond (green), cation–π interaction (magenta), π–sulfur interaction (yellow), σ-π interaction (gray), and π–π T-shaped interaction (orange).

**Figure 6:**
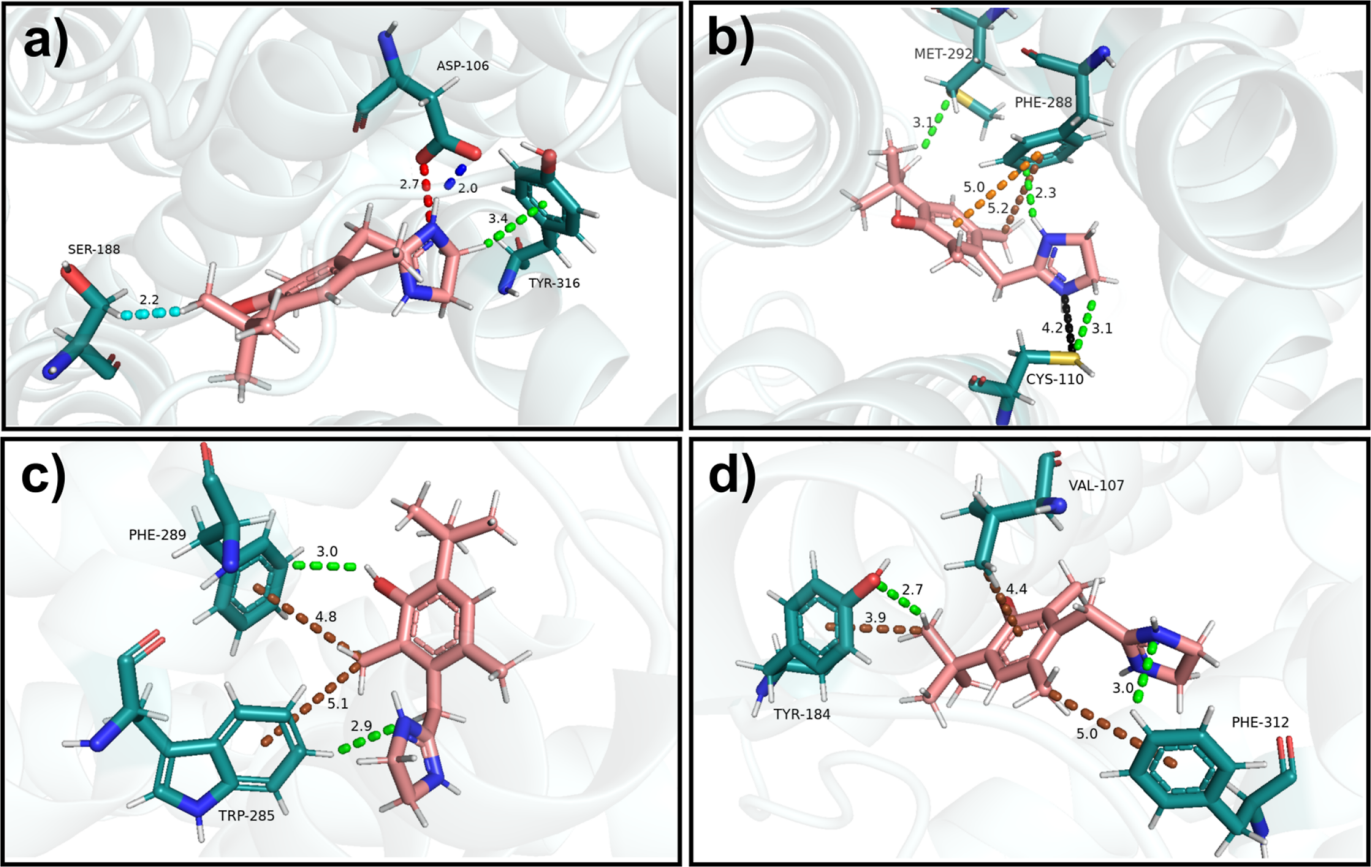
Visual representation of some of the main intermolecular interactions between oxymetazoline and the α_1A_-adrenoceptor. (a) Oxymetazoline interactions with ASP106, SER188, and TYR316. (b) Oxymetazoline interactions with CYS110, PHE288, and MET282. (c) Oxymetazoline interactions with TRP285 and PHE289. (d) Oxymetazoline interactions with VAL107, TYR184, and PHE312. Dashed lines represent different types of interactions: salt bridge (red), hydrogen bond (blue), nonconventional hydrogen bond (green), ion–dipole interaction (black), van der Waals interaction (cyan), π-alkyl interaction (brown), and π–π T-shaped interaction (orange).

**Figure 7:**
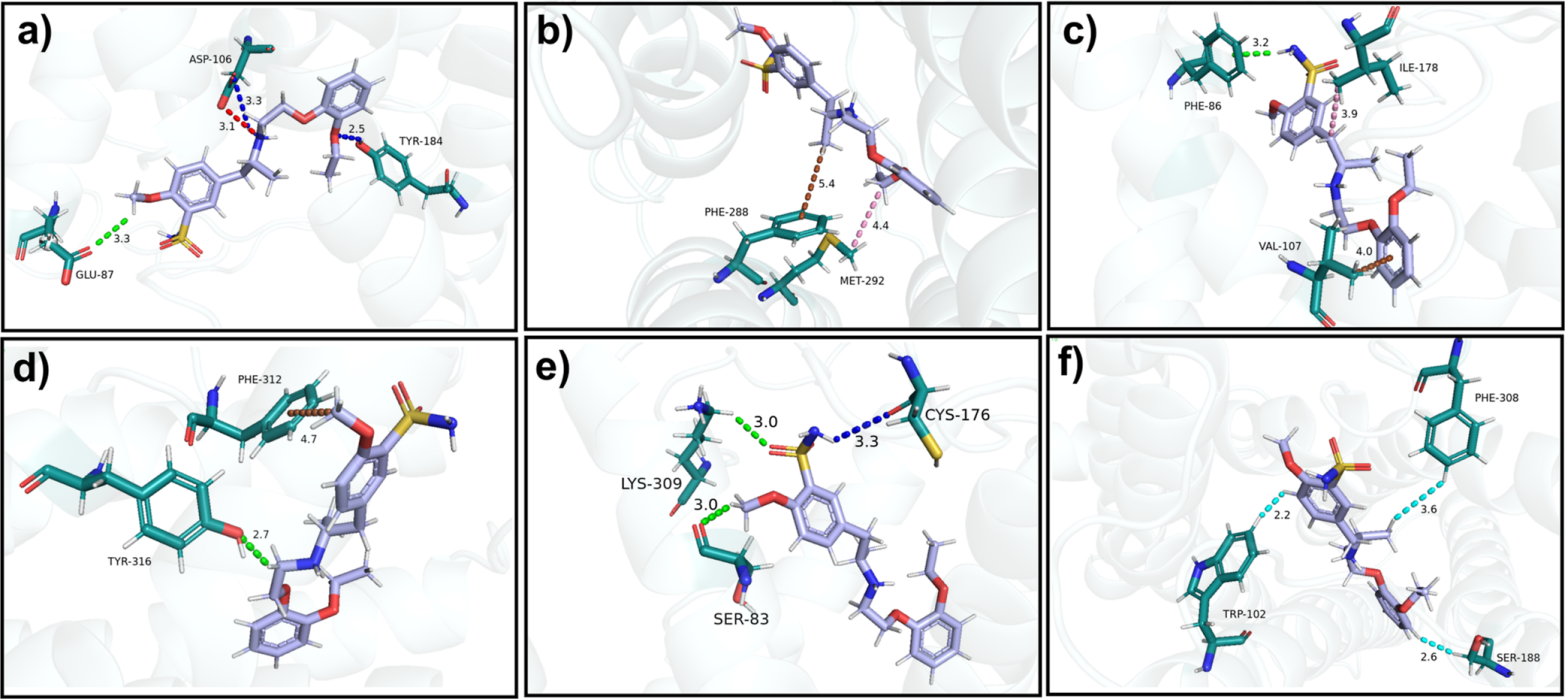
Visual representation of some of the main intermolecular interactions between tamsulosin and the α_1A_-adrenoceptor. (a) Tamsulosin interactions with GLU87, ASP106, and TYR184. (b) Interactions with PHE288 and MET292. (c) Interactions with PHE86, VAL107, and ILE178. (d) Interactions with PHE312 and TYR316. (e) Interactions with SER83, CYS176, and LYS309. (f) Interactions with TRP102, SER188, and PHE308. Dashed lines represent different types of interactions: salt bridge (red), hydrogen bond (blue), nonconventional hydrogen bond (green), van der Waals interactions (cyan), alkyl–alkyl interactions (pink), π-alkyl interaction (brown).

In terms of energetic values, the interactions of noradrenaline, oxymetazoline, and tamsulosin with the VAL107 residue were considered significant. Region i of noradrenaline interacts with VAL107 through σ-π interactions (Figure 5c) and van der Waals forces, with [i­(C4)­H] (2.09 Å) being the closest contact. Both oxymetazoline (region (ii) and tamsulosin (region (i) interact with VAL107 via π-alkyl interactions (Figures 6d and 7c) and extensive van der Waals forces, with [ii­(C8)­H] (2.29 Å) and [i­(C6)­H] (2.55 Å) representing the closest interactions for oxymetazoline and tamsulosin, respectively. The VAL107 residue is conserved among adrenoceptor subtypes. It is located near the α_1A_-AR binding pocket and plays a crucial role in proper ligand positioning through nonpolar and hydrophobic interactions.^5,14,19^ Crystallographic structures of the α_1A_-AR have shown that oxymetazoline, A61603, and tamsulosin interact with VAL107, whereas noradrenaline does not form such an interaction,^5^ an observation that differs from the findings of our study.

It is plausible to assume that geometry optimization performed on Cryo-EM structures may reveal noradrenaline-VAL107 interactions that are not apparent in the original structural data.^5^ This process relaxes atomic positions toward a local energy minimum, correcting minor steric clashes as well as bond lengths and angles. Such relaxation can lead to slight shifts in ligand or side-chain positions, potentially uncovering new close contacts or interactions (e.g., nonpolar interactions) that were not clearly resolved or fully formed in the original structure. As a result, the accuracy of the ligand-binding pose is improved, and subtle interactions relevant to ligand affinity may be highlighted.^48^

Noradrenaline, oxymetazoline, and tamsulosin showed some of their strongest interaction energies at PHE288 and PHE312 (Figure 4). Specifically, these residues ranked as the third and fourth strongest interactions for noradrenaline, the first and third for oxymetazoline, and the sixth and second for tamsulosin. To further investigate the molecular basis of these strong energetic interactions, we examined the nature of the intermolecular interactions established between each ligand and the residues PHE288 and PHE312. Region i of noradrenaline, represented by the catechol ring, engages in π–π T-shaped interactions with PHE288 (Figure 5b), while region ii forms π-cation interactions with PHE312 (Figure 5b). In the case of oxymetazoline, region ii establishes π–π T-shaped and π–alkyl interactions, as well as a nonconventional hydrogen bond [i­(N)­H] with PHE288 (Figure 6b), and further forms a π–alkyl interaction and an additional nonconventional hydrogen bond with PHE312 (Figure 6d). For tamsulosin, regions ii and iii are involved in π–alkyl interactions with PHE288 and PHE312, respectively (Figure 7b and d, respectively). In addition, van der Waals interactions were identified between each ligand and both PHE288 and PHE312, respectively, with the closest contacts identified as follows: [ii­(C8)­H] (2.19 Å) and [ii­(N)­H] (2.41 Å) for noradrenaline; [ii­(C1)­H] (2.29 Å) and [i­(C12)­H] (2.25 Å) for oxymetazoline; [i­(C2)­H] (2.59 Å) and [iii­(C27)­H] (2.17 Å) for tamsulosin. These observations are in line with previous studies demonstrating that both agonists (A601603, oxymetazoline, adrenaline, noradrenaline) and antagonists (tamsulosin, mirabegron) interact with PHE288 and PHE312 primarily through aromatic and polar interactions.^5,14,19^

Noradrenaline exhibited a repulsive interaction energy (1.40 kcal·mol^–1^) with residue MET292 (Figure 8), whereas oxymetazoline and tamsulosin showed attractive interaction energies (−3.08 and −1.58 kcal·mol^–1^, respectively). These favorable interactions can be attributed to the presence of nonconventional hydrogen bonds in the case of oxymetazoline (Figure 6b), alkyl–alkyl interactions for tamsulosin (Figure 7b), and van der Waals forces, with the closest contacts identified as [iii­(C17)­H] (2.23 Å) for oxymetazoline and [i­(C2)­H] (2.55 Å) for tamsulosin. MET292 is specific to the α_1A_-AR and is not conserved among other adrenoceptor subtypes. This unique residue may play a decisive role in the binding selectivity of ligands for α_1A_-AR over other adrenoceptors. Interactions involving MET292 have been reported in crystallographic structures with the adrenaline and selective α_1A_-AR ligands oxymetazoline, A61603, and tamsulosin.^5,14^ Additionally, similar interactions were observed in a homology model of the α_1A_-AR with doxazosin and an imidazoline derivative (*N*,*N*′-Bis­(tert-butoxycarbonyl)­imidazoline-2-thione).^49^ However, in the case of noradrenaline, the para-hydroxyl group of the catechol ring (region (i) is positioned near MET292, but does not engage in any polar interaction.^5^

**Figure 8:**
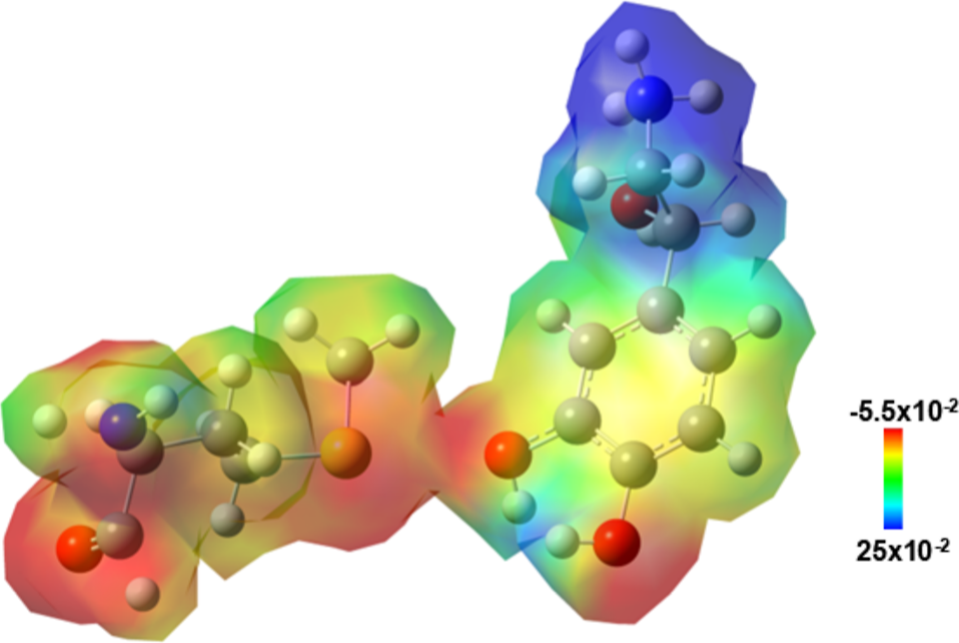
Isolated-state electrostatic potential (ESP) map derived from DFT of the norepinephrine–MET292 interface. Norepinephrine is shown on the right, and the MET292 side chain is on the left. The ESP is mapped onto the molecular electron density isosurface, with the color scale indicating regions of positive (blue) to negative (red) electrostatic potential. The map highlights electrostatic repulsion between the highly electronegative catechol ring of norepinephrine and the sulfur atom of the methionine side chain.

The repulsive interaction that occurs between norepinephrine and the MET292 residue (Figure 8)­takes place near the region of norepinephrine that contains the catechol ring and the sulfur-containing side chain of methionine. The proximity between the electron-rich oxygen atoms of the catechol portion and the sulfur atom of MET292 generates an unfavorable electronic overlap, resulting in local electrostatic repulsion. Since both atoms have high electron density and partial negative charge, the interaction is repulsive. This structural observation is further supported by the electrostatic potential map (ESP) generated from DFT calculations (Figure 8), which shows a region of high electron density (reddish tone) at the interface between the region i of noradrenaline and the thioether of MET292. Electrostatic repulsion at this site likely disrupts or prevents favorable polar contacts, reducing interaction stability.

It has been demonstrated that the substitution of LEU314 in α_1B_-AR (residue homologous to MET292 in the α_1A_-AR) with methionine significantly increases the binding affinity of the α_1A_-AR selective agonist oxymetazoline, while having no effect on the binding profile of the nonselective agonist noradrenaline.^50^ This finding reinforces the role of MET292 in mediating ligand selectivity at the α_1A_-AR. Furthermore, nuclear magnetic resonance (NMR) studies for ^13^C^ε^H_3_–methionine labeled α_1A_-AR mutants reveal that noradrenaline and oxymetazoline differentially modulate receptor conformations and activation states. Noradrenaline induces stronger conformational changes consistent with full activation, while oxymetazoline elicits partial changes reflecting its partial agonist nature.^51^

We propose that the repulsive interaction energy of noradrenaline with MET292 induces conformational strain or subtle rearrangements within the receptor binding pocket, which is consistent with the larger chemical shift perturbations observed for MET292 and adjacent residues in NMR experiments. In contrast, the attractive interaction of oxymetazoline stabilizes the ligand–receptor complex without imposing significant conformational strain, as reflected by the smaller chemical shift changes detected for MET292 in the presence of oxymetazoline.^51^ It is noteworthy that, in addition to exhibiting attractive interaction energy with MET292, tamsulosin interacts with the unique α_1A_-AR residue PHE86, as well as with several partially conserved residues (GLU87, TRP102, ILE178, and PHE312), which collectively position tamsulosin vertically within the binding pocket. This orientation enhances its selectivity for the α_1A_-AR and confers antagonistic activity by stabilizing the receptor in an inactive conformation.^5^

Other residues that are shared among noradrenaline, oxymetazoline, and tamsulosin and contribute significantly to their energetic interaction with the α_1A_-AR include CYS110, ILE178, SER188, PHE289, and TYR316 (Figure 4). Noradrenaline primarily forms a π–sulfur interaction with CYS110 (Figure 5c). Oxymetazoline interacts through a nonconventional hydrogen bond [i­(C12)­H] (3.10 Å) and an ion–dipole interaction [i­(NA)] (4.20 Å) (Figure 6b), while tamsulosin [i­(C8)­H] (2.62 Å) predominantly establishes van der Waals interactions with this residue. The CYS110 residue is conserved among α-ARs, but little information is available regarding its role in the interaction of noradrenaline and tamsulosin with the α_1A_-AR subtype. Structural studies have reported that imidazoline-type agonists, such as oxymetazoline and A61603, interact with CYS110 in the α_1A_-AR through nonpolar and hydrophobic interactions, respectively, suggesting that this residue may be important for ligand–receptor recognition of imidazoline compounds.^5,14^ In line with this suggestion, oxymetazoline exhibited a stronger energetic interaction with CYS110 (−2.51 kcal mol-1) compared to noradrenaline (−1.58 kcal mol-1) and tamsulosin (−1.38 kcal·mol^–1^) (Figure 4). Nevertheless, further structural and mutational studies are required to definitively establish the role of CYS110 in the differential ligand recognition at the α_1A_-AR.

For ILE178, noradrenaline interacts with this residue through a nonconventional hydrogen bond [ii­(O)] (3.40 Å) (Figure 5a), while oxymetazoline [ii­(C1)­H] (2.55 Å) and tamsulosin [ii­(C15)­H] (2.30 Å) form van der Waals interactions. ILE178 is a nonconserved residue uniquely found in the α_1A_-AR,^14^ whose role in ligand recognition at this receptor remains under debate. Structural data indicate that the α_1A_-selective agonist A61603 interacts with ILE178 primarily through hydrophobic contacts, highlighting the potential contribution of ILE178 to agonist binding and subtype selectivity.^14^ However, crystallographic structures do not reveal direct interactions between ILE178 and other ligands, such as the nonselective agonist’s adrenaline and noradrenaline, the selective α_1A_-AR agonist oxymetazoline, or the selective α_1A_-AR antagonist tamsulosin.^5,14^ On the other hand, mutagenesis studies have shown that residues GLN177, ILE178, and ASN179 contribute to the selectivity of antagonists such as phentolamine and WB4101 for α_1A_-AR over the α_1B_-AR subtype, suggesting that ILE178 may be determinant for antagonist binding.^52^ Consistently, the computed attractive interaction energy between tamsulosin and ILE178 (−3.65 kcal·mol^–1^) was greater than that observed for oxymetazoline (−1.99 kcal·mol^–1^) and noradrenaline (−1.27 kcal·mol^–1^) (Figure 4). However, whether ILE178 influences the binding affinity of nonselective agonists, selective agonists, or other antagonists at α_1A_-AR remains to be elucidated, particularly through mutagenesis studies.

The α_1A_-AR residues SER188, PHE289, and TYR316 are highly conserved among all ARs^5,14,51^ and contribute to ligand interactions and receptor function. For SER188, noradrenaline forms a H-bond [i­(OA)] (3.10 Å) (Figure 5a), whereas the closest contacts of oxymetazoline [iii­(C19)­H] (2.22 Å) and tamsulosin [i­(C5)­H] (2.59 Å) with this residue occur through van der Waals interactions (Figures 6a and 7f, respectively). Regarding PHE289, noradrenaline participates in a nonconventional H-bond [i­(OA)] (2.72 Å) (Figure 5c), oxymetazoline establishes both a nonconventional H-bond [ii­(O)­H] (3.0 Å) and a π-alkyl interaction (Figure 6c), while tamsulosin maintains van der Waals contacts. With TYR316, noradrenaline forms both a H-bond [i­(N)­H] (3.0 Å) and a salt bridge [i­(N)] (3.7 Å) (Figure 5b), whereas oxymetazoline [i­(C11)­H] (3.4 Å) and tamsulosin [i­(C12)­H] (2.7 Å) establish nonconventional H-bonds (Figures 6a and 7d, respectively).

It has been reported that the SER188 residue forms H-bonds with agonists such as adrenaline, noradrenaline, and the selective α_1A_-AR agonist A61603.^5,14^ Mutational studies demonstrated that substitution of SER188 with alanine in α_1A_-AR does not affect the binding affinity of agonists like adrenaline, phenylephrine, or synephrine but drastically reduces receptor activation. These findings indicate that the activation requirements of α_1A_-AR involve polar interactions mediated by SER188.^53^ Moreover, nonpolar interactions established by oxymetazoline and tamsulosin with this residue, as described here and elsewhere,^5^ could also contribute to the low efficacy of oxymetazoline, a partial agonist, and the neutral antagonism exerted by tamsulosin.

PHE289 is part of an aromatic residue network, including TRP285 and PHE288, that shapes the active site of the α_1A_-AR and contributes to ligand positioning and stabilization within the binding pocket.^14,19^ Consistent with our findings, crystallographic studies have demonstrated that PHE289 participates in nonpolar interactions with A61603, noradrenaline, and tamsulosin, while aromatic interactions are formed between PHE289 and oxymetazoline.^5,14^ Similarly, TYR316 appears to be important in ligand stabilization with the binding pocket of α_1A_-AR. In this study, we observed that noradrenaline forms polar interactions with this residue, while others have reported that adrenaline and A61603 establish nonpolar interactions, and oxymetazoline engages in aromatic interactions. Critically, TYR316 forms a hydrogen bond with ASP106, stabilizing the salt bridge between the protonated nitrogen of adrenergic ligands and ASP106, which is essential for ligand binding to the α_1A_-AR,^5,14^ as previously discussed.

GLU180, VAL185, and ALA189 are residues in the α_1A_-AR that are energetically important for the interaction with the selective partial agonist oxymetazoline. The *tert*-butyl group of this ligand (region (iii) primarily forms van der Waals interactions with GLU180, VAL185, and ALA189, with the closest contacts identified as [iii­(C18)­H] (3.67 Å), [iii­(C18)­H] (2.18 Å), and [iii­(C17)­H] (2.83 Å), respectively. Among these three residues, VAL185 appears to be essential for the binding of imidazoline-type agonists such as oxymetazoline and A61603 to the α_1A_-AR. Mutagenesis studies revealed that substituting VAL185 with alanine (VAL185A) leads to a reduced interaction of oxymetazoline with α_1A_-AR, without affecting noradrenaline binding.^54^ Molecular dynamics simulations performed on the α_1A_-AR-VAL185A mutant showed that the interaction between the imidazoline group of A61603 and ASP106 was compromised, which is determinant for the affinity and efficacy of various ligands at the α_1A_-AR. In contrast, mutation of ALA189 to serine (ALA189S) did not impair the interaction between A61603 and ASP106, with the functional response of A61603 remaining preserved upon α_1A_-AR-ALA189S activation.^14^ The importance of GLU180 in imidazoline-type ligand binding at the α_1A_-AR is still unclear and warrants further investigation.

Unlike noradrenaline, oxymetazoline contains a *tert*-butyl group (region (iii) that forms nonpolar interactions with residues such as VAL185 and ALA189, which are not established by noradrenaline. Additionally, oxymetazoline exhibits attractive interactions with MET292 but does not establish polar contacts with SER188. These distinct interaction patterns with VAL185, SER188, ALA189, and MET292 may stabilize a unique receptor conformation compared to noradrenaline, potentially explaining the biased agonism of oxymetazoline at α_1A_-AR toward β-arrestin recruitment, as previously described.^55,56^ Mutagenesis studies should be conducted to determine whether these residues indeed contribute to the distinct pharmacological profile of oxymetazoline.

The selective α_1A_-AR antagonist tamsulosin, but not the selective α_1A_-AR agonist oxymetazoline or the nonselective agonist noradrenaline, exhibited significant energetic interactions with residues SER83, PHE86, GLU87, TRP102, CYS176, PHE308, and LYS309. Nonconventional H-bonds were identified between the methoxybenzenesulfonamide group (region (iii) of tamsulosin and the residues SER83 (Figure 7e), PHE86 (Figure 7c), GLU187 (Figure 7a), and LYS309 (Figure 7e), involving specifically the atoms [iii­(C27)­H] (2.98 Å), [iii­(O)] (2.95 Å), [iii­(C27)­H] (2.98 Å), and [iii­(OC)] (3.03 Å), respectively. Region iii of tamsulosin, specifically [iii­(N)­H] (3.30 Å), also established a H-bond with CYS176 (Figure 7e), as well as van der Waals interactions with TRP102 (Figure 7f), with the closest contact involving [iii­(C18)­H] (2.15 Å). On the other hand, the ethylaminopropyl group (region (ii) of tamsulosin forms van der Waals interactions with PHE308 (Figure 7f), with [ii­(C28)­H] representing the closest contact at 3.59 Å.

Region iii of tamsulosin extends toward the extracellular vestibule of the α_1A_-AR, enabling interactions with the unique residue PHE86, the partially conserved residues SER83, GLU87, TRP102, and LYS309, and the conserved residue CYS176. These interactions contribute to the enhanced selectivity of tamsulosin for the α_1A_-AR compared to other ligands, such as the agonists oxymetazoline and noradrenaline.^5^ In addition, it is reported that PHE308 is a key determinant of antagonist binding affinity in the α_1A_-AR. Mutagenesis studies have demonstrated that substitution of this residue leads to a significant reduction in the binding affinity of several antagonists, including prazosin, WB4101, BMY7378, (+)-niguldipine, and 5-methylurapidil.^20^ Moreover, mutation of PHE308 in the α_1A_-AR also reduces the affinity of oxymetazoline, although to a lesser extent compared to the PHE312 mutation.^20^ In line with this, our analysis revealed that oxymetazoline interacts with PHE308 through van der Waals forces, with the closest contact involving [ii­(C1)­H] at 5.27 Å and a low energetic contribution of −0.32 kcal·mol^–1^ (Supporting Information).

## Conclusions

This study used a quantum biochemistry approach that combined Density Functional Theory (DFT) with the Molecular Fractionation with Conjugate Caps (MFCC) method to characterize the molecular interactions between the α_1A_-AR and three ligands, noradrenaline, oxymetazoline, and tamsulosin. The analysis identified key residues, particularly ASP106, VAL107, PHE288, and PHE312, as major contributors to the total binding energy, consistent with available structural and mutagenesis data. A comparative assessment revealed recurring interaction patterns across the ligands, hydrogen bonding with ASP106 and π–π interactions with PHE288 and PHE312 were the dominant stabilizing forces within the binding pocket. These interactions largely determine both affinity and selectivity profiles, explaining the distinct pharmacological behaviors of each compound. The binding energy profiles of noradrenaline, oxymetazoline, and tamsulosin accurately reflected their experimental affinity ranking, further supporting the reliability of the MFCC–DFT protocol for modeling ligand–receptor interactions. Recognition of these dominant interaction motifs provides valuable guidance for the rational design of more efficient and selective α_1A_-AR ligands, particularly through strategic reinforcement of hydrogen-bonding capacity near ASP106 and optimization of aromatic stacking with PHE residues. These findings establish a robust theoretical basis to guide the development of next-generation α_1A_-AR ligands with improved efficacy and safety profiles. Future design efforts should focus on selecting substituents that strengthen key polar and aromatic interactions while preserving the hydrophobic complementarity of the binding site. These structure-based strategies can accelerate the discovery of α_1A_-AR modulators with refined selectivity, reduced off-target effects, and optimized pharmacodynamic properties. Further experimental validation through site-directed mutagenesis and structure–activity relationship studies is needed to confirm the functional relevance of the identified residues.

## Supplementary Material



## Data Availability

The data supporting this study, including the input files for the MFCC and DFT calculations, can be accessed at https://zenodo.org/records/15707365. Additional data sets are provided in the Supporting Information. Other data have also been included as part of the Supporting Information.

## Notes

The following files are available free of charge. The Supporting Information is available free of charge at https://pubs.acs.org/doi/10.1021/acsomega.5c08861.

The Supporting Information.pdf includes Tables S1–S3, which present the complete results of the energy calculations, along with the atomic group closest in distance and the interaction radius corresponding to each residue (PDF)
