# Supplementary material for: Quantum Biochemistry Insights into Ligand Recognition at the a1A-Adrenoceptor
Source: ACS Omega. 2026 Feb 11;11(7):11403–16. doi: 10.1021/acsomega.5c08861 (PMC12947219; doi:10.1021/acsomega.5c08861)
Supplement: Supplementary file 1 [file ao5c08861_si_001.pdf]

Supporting Information

Quantum biochemistry insights into ligand recognition at the α_{1A}-adrenoceptor

Luana Talinne da Costa Gomes[1], Katyanna Sales Bezerra[2], Elaine Cristina Gavioli[1], Jonas Ivan Nobre de Oliveira[1], Douglas Soares Galvão[2], Umberto Laino Fulco*[1], Edilson Dantas da Silva Junior[1]

[1] Department of Biophysics and Pharmacology, Federal University of Rio Grande do Norte, , Natal, RN, Brazil.

[2] Applied Physics Department, University of Campinas, 130838-59, Campinas, São Paulo, Brazil.

*Corresponding author (ULF)
Department of Biophysics and Pharmacology, Federal University of Rio Grande do Norte, Av. Senador Salgado Filho, s/n Campus Universitário – Lagoa Nova, Natal 59072-970, RN, Brazil. Tel.: +55 84 3215-3419. Electronic address: umbertofulco@gmail.com

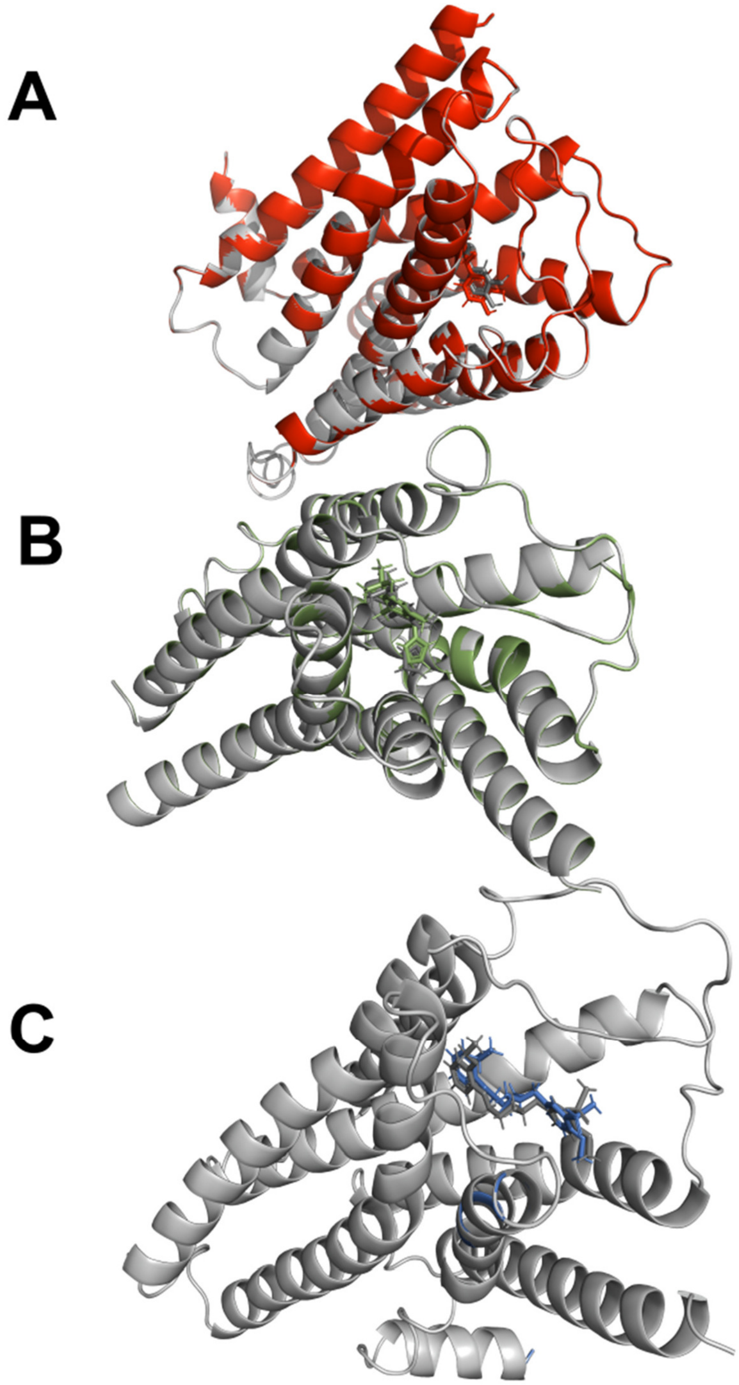

Figure S1: Structural overlap of the three protein structures used in this study before and after energy minimization. The RMSD values calculated between the pre- and post-minimization structures were 0.180 Å for PDB ID: 7YMH (A), 0.168 Å for PDB ID: 7YM8 (B), and 0.178 Å for PDB ID: 7YMJ (C), indicating minimal conformational deviations and preservation of overall folding.

Table S1. Interaction of noradrenaline with the α_{1A}-adrenoceptor: involved amino acid residues, ligand atomic groups, interaction radius, distances, and energy values (in kcal/mol) for different dielectric constants (ε=10 and ε=40).

Amino Acid	*Atomic group*	*Radius (Å)*	*Distance (Å)*	*ε=10*	*ε=40*
ASP106	ii(N)H	2	1.80	-16.12	-8.33
VAL107	i(C4)H	2.5	2.09	-4.72	-4.50
CYS110	i(C4)H	2.5	2.18	-1.74	-1.58
PHE288	ii(C8)H	2.5	2.19	-3.40	-3.36

PHE312	ii(N)H	2.5	2.41	-4.15	-3.19
ILE178	i(C6)H	3	2.65	-0.94	-1.27
VAL185	i(O_A)H	3	2.75	-0.86	-0.74
SER188	i(O_B)H	3	2.89	-0.81	-0.84
PHE289	i(O_B)H	3	2.59	-1.92	-1.89
MET292	i(C6)H	3	2.65	1.43	1.40
TYR316	ii(N)H	3	2.97	-1.56	-1.57
THR111	i(C3)H	3.5	3.07	-0.63	-0.71
TYR184	ii(O_A)H	3.5	3.38	-1.01	-1.04
TRP285	ii(C8)H	3.5	3.40	-1.06	-0.82
ALA189	i(O_B)H	4	3.96	-0.42	-0.39
ALA103	ii(O)H	4.5	4.08	-1.21	-0.64
SER192	i(C3)H	4.5	4.21	-0.64	-0.57
LEU75	ii(N)H	5	4.55	-0.24	-0.22
VAL79	ii(N)H	5	4.51	-0.14	-0.18
TRP102	ii(N)H	5	4.90	-0.75	-0.49
ILE114	i(C3)H	5	4.56	-0.01	-0.15
ILE157	i(C3)H	5	4.53	-0.43	-0.44
ASN179	ii(O)H	5	4.91	-0.25	-0.30
SER83	ii(N)H	5.5	5.45	0.16	0.00
LEU108	i(C4)H	5.5	5.14	-0.13	-0.18
GLU180	i(O_A)	5.5	5.26	-3.42	-1.01
GLY315	ii(C8)H	5.5	5.14	0.22	-0.02
CYS109	i(C4)H	6	5.91	0.53	0.16
PRO293	i(O_B)H	6	5.70	0.01	-0.03
PHE308	i(C6)H	6	5.96	-0.37	-0.19
LEU80	ii(N)H	6.5	6.31	-0.21	-0.09
ALA104	ii(O)H	6.5	6.22	-0.44	-0.19
VAL105	ii(C7)H	6.5	6.02	0.47	0.29
PHE193	i(O_B)H	6.5	6.19	-0.13	-0.12
TRP313	ii(N)H	6.5	6.14	-0.33	-0.17
ALA112	i(C4)H	7	6.55	0.15	0.02
SER158	i(C3)H	7	6.63	-0.17	-0.12
LEU190	i(O_B)H	7	6.91	-0.10	-0.06
VAL311	ii(C8)H	7	6.57	-0.03	0.01
SER154	i(C3)H	7.5	7.4	-0.15	-0.06
PRO161	i(O_A)H	7.5	7.48	-0.02	-0.03
GLU181	i(O_A)H	7.5	7.29	-2.17	-0.56
LEU186	i(O_A)H	7.5	7.004	-0.17	-0.08
PHE187	i(O_B)	7.5	7.08	-0.02	-0.09
SER113	i(C4)H	8	7.58	0.24	0.04
MET115	i(C3)H	8	7.81	0.13	0.02
GLN177	ii(O)H	8	7.59	-0.07	-0.04
TYR194	i(O_B)H	8	7.99	0.07	0.00

SER296	$i(O_A)H$	8	7.95	0.00	-0.01
GLY191	$i(O_B)$	8.5	8.04	0.05	0.00
LYS309	ii(N)H	8.5	8.42	2.37	0.58
LEU314	ii(N)H	8.5	8.09	0.17	0.02
LEU317	ii(N)H	8.5	8.37	0.07	0.01
PHE86	ii(N)H	9	8.76	0.13	0.02
CYS99	ii(O)H	9	8.59	-0.25	-0.07
ILE101	ii(O)H	9	8.89	-0.11	-0.04
LEU153	i(C3)H	9	8.60	-0.04	-0.02
CYS176	ii(O)H	9	8.65	-0.21	-0.07
PRO182	$i(O_A)H$	9	8.61	-0.04	-0.02
PRO196	i(C3)H	9	8.78	0.10	0.02
LEU290	$i(O_B)H$	9	8.63	0.05	0.00
VAL291	$i(O_A)$	9	8.93	0.09	-0.07
ASN318	ii(C8)H	9	8.69	0.37	0.09
SER319	ii(C8)H	9	8.70	-0.05	-0.02
THR76	ii(N)H	9.5	9.42	0.01	-0.01
PHE82	ii(N)H	9.5	9.30	0.21	0.04
TRP92	ii(N)H	9.5	9.33	-0.05	-0.02
GLY183	$i(O_A)H$	9.5	9.29	0.09	0.02
PHE281	i(C3)H	9.5	9.40	-0.17	-0.06
CYS284	ii(C8)H	9.5	9.28	0.03	0.01
ILE310	ii(N)H	9.5	9.47	-0.14	-0.05
ASP72	ii(C8)H	10	9.89	-2.95	-0.75
ALA84	ii(N)H	10	9.85	-0.02	-0.01
ASN100	ii(O)H	10	9.50	-0.26	-0.08
LEU155	i(C3)H	10	9.86	-0.12	-0.04
VAL156	i(C3)H	10	9.85	0.00	-0.02
ILE159	i(C3)H	10	9.84	0.00	-0.02
GLY160	$i(O_B)$	10	9.65	0.19	0.04
TRP165	$i(O_A)H$	10	9.85	0.01	-0.01
ARG166	ii(O)H	10	9.59	2.55	0.63
PRO287	ii(C8)H	10	9.69	0.17	0.06
ILE294	$i(O_A)$	10	9.67	0.09	0.02

Table S2. Interaction of oxymetazoline with the α_{1A}-adrenoceptor: involved amino acid residues, ligand atomic groups, interaction radius, distances, and energy values (in kcal/mol) for different dielectric constants (ε=10 and ε=40).

Amino Acid	*Atomic group*	*Radius (Å)*	*Distance (Å)*	*ε=10*	*ε=40*
ASP106	i(N_B)H	2	1.96	-13.31	-6.88
VAL107	ii(C8)H	2.5	2.29	-4.92	-4.68
THR111	ii(C14)H	2.5	2.42	-1.10	-1.17
TYR184	iii(C18)H	2.5	2.11	-2.88	-2.77
VAL185	iii(C18)H	2.5	2.18	-1.98	-1.75
SER188	iii(C19)H	2.5	2.22	-1.26	-1.39
PHE288	i(N_A)H	2.5	2.28	-8.11	-7.83
PHE289	ii(C14)H	2.5	2.29	-2.69	-2.54
MET292	iii(C17)H	2.5	2.23	-2.87	-3.08
PHE312	i(C12)H	2.5	2.25	-6.70	-5.93
CYS110	ii(C8)H	3	2.67	-2.46	-2.51
ILE178	ii(C1)H	3	2.55	-1.73	-1.99
ASN179	iii(C18)H	3	2.83	-0.63	-0.40
ALA189	iii(C17)H	3	2.83	-1.38	-1.33
TRP285	i(C12)H	3	2.54	-4.39	-3.93
GLY315	i(C11)H	3.5	3.14	-0.17	-0.49
TYR316	i(C12)H	3.5	3.33	-2.24	-2.33
ILE157	iii(C19)H	4	3.86	-0.81	-0.84
GLU180	iii(C18)H	4	3.67	-3.81	-1.37
SER192	ii(C14)H	4	3.61	-0.60	-0.66
LEU75	i(N_B)H	4.5	4.39	-0.36	-0.35
ILE114	ii(C14)H	4.5	4.10	-0.30	-0.44
GLU181	iii(C18)H	4.5	4.48	-2.27	-0.69
VAL79	i(N_B)H	5	4.81	0.01	-0.10
LEU186	iii(C17)H	5	4.82	-0.34	-0.22
VAL311	i(C11)H	5	4.71	-0.33	-0.20
ALA103	ii(C8)H	5.5	5.03	-0.74	-0.37
LEU108	ii(C8)H	5.5	5.49	-0.15	-0.17
PHE193	ii(C14)H	5.5	5.30	-0.21	-0.20
PRO293	iii(C17)H	5.5	5.08	-0.19	-0.21
PHE308	ii(C1)H	5.5	5.27	-0.56	-0.32
TRP313	i(C12)H	5.5	5.13	-0.51	-0.33
SER83	i(C12)H	6	5.82	0.17	0.00
SER158	iii(C19)H	6	5.69	-0.18	-0.17
PRO161	iii(C19)H	6	5.60	-0.03	-0.13
PHE187	iii(C17)H	6	5.89	-0.15	-0.20
LEU190	iii(C17)H	6	5.63	-0.20	-0.11
LEU314	i(C11)H	6	5.98	0.18	-0.04
LEU80	i(C12)H	6.5	6.19	-0.16	-0.10
CYS109	ii(C8)H	6.5	6.39	0.37	0.02
CYS284	i(C11)H	6.5	6.45	-0.09	-0.05
SER296	iii(C17)H	6.5	6.15	-0.07	-0.07
TRP102	i(N_B)H	7	6.76	-0.47	-0.25
ALA104	ii(C8)H	7	6.77	-0.36	-0.16
VAL105	ii(C8)H	7	6.76	0.19	0.09
ALA112	ii(C14)H	7	6.88	0.09	0.00
GLY160	iii(C19)H	7	6.74	0.16	0.02
PRO182	iii(C18)H	7	6.92	-0.03	-0.05

GLY183	iii(C18)H	7	6.53	0.07	-0.04
LEU317	i(C12)H	7	6.87	0.12	0.02
SER113	ii(C14)H	7.5	7.32	0.22	0.03
MET115	ii(C14)H	7.5	7.01	0.08	-0.02
SER154	ii(C14)H	7.5	7.26	-0.12	-0.07
TRP165	iii(C19)H	7.5	7.21	-0.17	-0.10
GLN177	ii(C1)H	7.5	7.19	0.04	-0.03
GLY191	iii(C17)H	7.5	7.23	0.05	-0.03
LYS309	i(C11)H	7.5	7.40	2.29	0.54
ASN318	i(C12)H	7.5	7.24	0.37	0.07
SER319	i(C12)H	7.5	7.41	-0.04	-0.03
CYS176	ii(C1)H	8	7.98	-0.14	-0.06
TYR194	iii(C17)H	8	7.62	0.04	-0.03
PHE281	ii(C14)H	8	7.82	-0.23	-0.09
LEU290	iii(C17)H	8	7.88	0.05	-0.02
VAL291	iii(C17)H	8	7.77	0.19	-0.01
ILE310	i(C12)H	8	7.78	-0.16	-0.06
ILE159	iii(C19)H	8.5	8.37	0.00	-0.02
ARG166	iii(C18)H	8.5	8.13	2.31	0.54
PRO196	ii(C14)H	8.5	8.48	0.11	0.01
PRO287	i(C11)H	8.5	8.22	0.25	0.10
ILE294	iii(C17)H	8.5	8.03	0.08	0.00
PHE297	iii(C17)H	8.5	8.13	0.01	-0.02
LEU153	ii(C14)H	9	8.98	-0.04	-0.03
VAL156	iii(C19)H	9	8.53	0.01	-0.02
LEU286	i(C11)H	9	8.91	-0.17	-0.07
ASP72	i(C12)H	9.5	9.34	-2.91	-0.74
THR76	i(C12)H	9.5	9.16	0.02	0.00
PHE86	ii(C1)H	9.5	9.25	0.11	0.01
GLY295	iii(C17)H	9.5	9.03	0.12	0.02
ILE37	i(C12)H	10	9.97	-0.05	-0.02
PHE82	i(N_B)H	10	9.90	0.15	0.03
ALA84	i(C12)H	10	9.83	-0.03	-0.01
TRP92	i(N_B)H	10	9.98	-0.05	-0.02
CYS99	ii(C1)H	10	9.64	-0.16	-0.05
ILE101	ii(C8)H	10	9.77	-0.12	-0.04
LEU162	iii(C19)H	10	9.55	0.01	-0.01
GLN167	iii(C18)H	10	9.74	-0.08	-0.03
LEU195	ii(O)H	10	9.87	0.10	0.02
VAL307	i(C11)H	10	9.51	-0.10	-0.03
CYS320	i(C12)H	10	9.84	0.04	0.01

Table S3. Interaction of tamsulosin with the α_{1A}-adrenoceptor: involved amino acid residues, ligand atomic groups, interaction radius, distances, and energy values (in kcal/mol) for different dielectric constants (ε=10 and ε=40).

Amino Acid	*Atomic group*	*Radius (Å)*	*Distance (Å)*	*ε=10*	*ε=40*
SER83	iii(C27)H	2.5	2.27	-1.52	-1.54
TRP102	iii(C18)H	2.5	2.15	-2.47	-2.13
ASP106	ii(N)H	2.5	2.15	-22.41	-14.59
ILE178	ii(C15)H	2.5	2.30	-3.47	-3.65
TYR184	i(C1)H	2.5	2.21	-3.09	-3.03
PHE312	iii(C27)H	2.5	2.17	-5.45	-5.08
TYR316	i(C12)H	2.5	2.40	-1.91	-1.83
PHE86	iii(N)H	3	2.502	-3.26	-3.39
GLU87	iii(C27)H	3	2.98	-3.00	-1.43
VAL107	i(C6)H	3	2.55	-4.95	-4.65
CYS110	i(C8)H	3	2.62	-1.29	-1.38
THR111	i(C7)H	3	2.99	-0.75	-0.82
SER188	i(C5)H	3	2.59	-1.46	-1.41
PHE288	i(C2)H	3	2.59	-2.79	-2.78
MET292	i(C2)H	3	2.55	-1.66	-1.58
TRP313	iii(C27)H	3	2.62	-1.37	-1.25
CYS176	iii(N)H	3.5	3.33	-1.00	-0.75
SER192	i(C6)H	3.5	3.36	-1.03	-1.02
LYS309	iii(O_C)	3.5	3.03	-0.54	-1.48
GLN177	iii(N)H	4	3.93	-0.32	-0.42
TRP285	i(C12)H	4	3.79	-1.00	-0.84
PHE289	i(C7)H	4	3.60	-1.11	-1.05
PHE308	ii(C28)H	4	3.59	-1.52	-1.35
ASN179	i(C1)H	4.5	4.47	-0.79	-0.42
ALA84	iii(C27)H	5	4.95	-0.22	-0.19
ALA103	ii(N)H	5	4.93	-0.66	-0.40
ILE114	i(C7)H	5	4.62	-0.02	-0.13
SER158	i(C6)H	5	4.77	-0.48	-0.31
VAL79	i(C12)H	5.5	5.32	-0.22	-0.24
VAL185	i(C1)H	5.5	5.07	-0.38	-0.27
LEU75	i(C12)H	6	5.82	-0.17	-0.14
VAL79	i(C12)H	6	5.32	-0.22	-0.24
PHE82	iii(C27)H	6	5.75	0.13	-0.05
LEU108	i(C7)H	6	5.68	-0.21	-0.23
ILE157	i(C6)H	6	5.97	-0.12	-0.10
PRO161	i(C5)H	6	5.75	0.03	-0.06
LEU162	i(C1)H	6	5.75	-0.21	-0.20
GLU180	i(C1)H	6	5.79	-2.89	-0.82
ALA189	i(C6)H	6	5.96	-0.20	-0.17
LEU80	iii(C18)H	6.5	6.17	-0.32	-0.19
VAL88	iii(C27)H	6.5	6.15	0.03	-0.02
VAL105	i(C11)H	6.5	6.23	0.37	0.09
CYS109	i(C7)H	6.5	6.39	0.32	0.01
GLY191	i(C6)H	6.5	6.38	0.09	-0.03
PHE193	i(C7)H	6.5	6.40	-0.12	-0.11
ILE85	iii(C27)H	7	6.58	0.12	0.01
TRP92	iii(N)H	7	6.53	-0.17	-0.16
ALA112	i(C7)H	7	6.54	0.09	0.00

PHE187	i(C6)H	7	6.96	0.04	-0.05
LEU33	iii(C27)H	7.5	7.13	-0.06	-0.04
GLY90	iii(N)H	7.5	7.09	0.05	-0.01
TYR91	iii(N)H	7.5	7.11	0.01	-0.03
ALA104	i(C11)H	7.5	7.40	-0.14	-0.11
ILE175	iii(N)H	7.5	7.29	0.10	-0.02
GLU305	iii(O_D)	7.5	7.31	-1.72	-0.46
VAL311	ii(C28)H	7.5	7.02	-0.08	-0.12
GLY315	i(C12)H	7.5	7.05	0.15	0.01
PRO81	iii(C27)H	8	7.88	-0.05	-0.05
ARG113	i(C7)H	8	7.54	2.46	0.59
TRP115	i(C7)H	8	7.65	0.14	0.01
ILE159	i(C6)H	8	7.73	-0.06	-0.06
GLU181	i(C1)H	8	7.98	-2.58	-0.67
LEU190	i(C6)H	8	7.69	0.03	-0.02
PRO293	i(C2)H	8	7.63	-0.04	-0.04
SER296	i(C1)H	8	7.99	0.02	-0.01
LEU89	iii(C27)H	8.5	8.15	0.08	0.00
CYS99	iii(N)H	8.5	8.15	-0.40	-0.14
SER154	i(C6)H	8.5	8.18	-0.07	-0.03
GLY160	i(C6)H	8.5	8.48	0.08	0.01
LEU186	i(C2)H	8.5	8.31	-0.11	-0.05
PHE281	i(C7)H	8.5	8.15	-0.13	-0.05
ILE310	iii(C27)H	8.5	8.13	-0.11	-0.07
LEU29	iii(C27)H	9	8.83	-0.06	-0.03
ILE37	iii(C27)H	9	8.99	-0.01	-0.01
TYR194	i(C6)H	9	8.87	0.05	-0.01
PRO196	i(C7)H	9	8.86	0.10	0.02
VAL291	ii(C28)H	9	8.66	0.14	0.01
SER319	i(C11)H	9	8.94	0.11	0.02
ASN100	ii(C15)H	9.5	9.44	-0.35	-0.11
TRP165	i(C1)H	9.5	9.03	-0.08	-0.04
THR174	iii(N)H	9.5	9.02	0.02	0.00
LYS302	iii(O_D)	9.5	9.21	1.81	0.44
THR306	iii(O_C)	9.5	9.07	-0.04	-0.02
LEU314	i(C12)H	9.5	9.49	0.12	0.02
ALA93	iii(N)H	10	9.98	0.05	0.01
ARG96	iii(N)H	10	9.57	1.99	0.49
ILE101	ii(N)H	10	9.53	-0.11	-0.08
LEU155	i(C6)H	10	9.86	-0.12	-0.04
VAL156	i(C6)H	10	9.63	-0.06	-0.02
PRO182	i(C1)H	10	9.76	-0.12	-0.04
GLY183	i(C5)H	10	9.58	0.02	-0.01
LEU195	i(C6)H	10	9.81	0.11	0.02
SER304	iii(O_D)	10	9.68	-0.13	-0.04
ASN318	i(C8)H	10	9.72	0.32	0.07
